# A Case of Canine Tuberculosis

**Published:** 1896-05

**Authors:** Frank Miller

**Affiliations:** Canine Veterinary Academy, Berlin


### REPORTS OF CASES.

### A CASE OF CANINE TUBERCULOSIS.

By FRANK MILLER, V.S.,
CANINE VETERINARY ACADEMY, BERLIN.

A DOG was brought to the polyclinic for small animals in the Veterinary Academy at Berlin, February 1, 1896, and admitted to the internal clinic with the following history and result:

The patient is said to have been in poor health for several months, and had been treated before in the polyclinic. At times the medicine seemed to benefit the patient, at other times it was without effect. The owner gave want of appetite, great thirst, coughing, and blood in the urine, as cardinal symptoms noted by him.

Systematic physical examination was as follows: The patient, a hound seven years of age, is in extremely poor condition of flesh. The bones are prominent. The vertebral column is roached. The hair is dull. The temperature is evenly distributed and is 40.4° C. in the rectum. Pulse 110 per minute, full and rhythmic. The heart-tones are indistinct and are replaced by murmur. The heart-beat is to be felt on both sides. Respiration, 20 per minute. The patient coughs, particularly during percussion. The cough is weak and toneless. The percussion gives a loud clear tone, and auscultation gives a vesicular respiratory sound. The respiration is deep, and the thoracic frame is active. Inspiration and expiration bear the proper relation to each other. The appetite is very poor and thirst much marked. The abdomen is tucked up, or contracted. Palpation of the region of the stomach and kidneys causes uneasiness. The palpation of the bowels and bladder reveals nothing abnormal. Rectal examination shows the mucous membrane swollen. The patient has marked tenesmus. The intestinal evacuations are semifluid and dark-brown in color. Urine was not observed. The movements are slow and faltering. There is entropium of both upper eyelids, and crusts of semi-dried mucus in the internal canthii. The conjunctival membranes are of pale washy-red color. The eyes, clear, with complaining expression. Psyche depressed. The average temperature, pulse, and respiration while under observation was as follows: Temperature, 39.55° C.; pulse, 118; respiration, 24.

Patient failed in condition gradually, and died February 19th under symptoms of collapse. The report from the Pathological Institute, where post-mortem was made, reads as follows:

"No. 1841. Cadaver of emaciated dog. Rigor mortis present. Pleura costalis, pericardialis, and diaphragmatica thickened and covered with multitudinous round, warty, pea-sized nodules, which in a few cases by conglomerating gave small apple-sized nodes. In sections these nodules were smooth and gray and white in color, of semi-solid consistency (cheesy). The anterior and posterior mediastinal glands are enlarged and tumefied, and in the centre are caseous. In the centre of the mediastinum is a tumor of fist size, whose centre is entirely broken down into an abscess, the walls of which are thick, and contains yellowish-white, thin material, which, microscopically examined, consisted of broken-down glandular tissue of primarily tuberculous nature and mixed infection. The pulmonary pleura is smooth and glistening, lungs marked with black pigment and well inflated; however, much serum escapes on cutting. The pericardium contains a few grammes of yellowish flocculent fluid. The auricles and ventricles of the heart are slightly dilated without compensatory thickening. The valves are normal, as is also the entire endocardium. The peritoneum is smooth and pink in color. The small intestines contain liquid contents. The intestinal mucous membrane and that of the glandular section of the stomach is of bluish-green hue and covered with mucous. The spleen is enlarged and of blue-red color; its pulp is firm and its trabecular structure is visible in its section. The liver is somewhat enlarged, of deep red color, and is filled with blood which flows freely from its cut surface. The mucous membrane of the bladder is studded with gray-black nodules of pea-size, and is also marked by small hemorrhagic spots of pin-head-size. The renal capsules are clearly adherent to the kidneys, which are nodular and of gray-red appearance. Many small grayish-yellow nodules are seen upon their surface, to which they give the peculiar uneven contour mentioned. In places they aggregated into patches of dollar-size. Upon section these spots are seen to dip down from the cortex toward the pelvis in wedge form, but are not sharply defined from the surrounding tissues. Microscopic examination gave tuberculous tissue and bacilli Kochii.

"Pathological diagnosis: Acute general tuberculosis. Gastric contents, catarrhal. Œdema pulmonum. Dilatation of cardiac chambers. Cystitis hemorrhagica."

This case has many interesting points as written. In addition to the hospital report, I wish to mention that I have ascertained that the patient had received 0.02 tuberculin hypodermically about three months prior to his admission. This failed to give any reaction. The conditions found at the post-mortem, at least some of them, indicated that the process had been going on for several months, especially in the mediastinal glands. The condition of calcification was entirely absent, as is usual in canine tuberculosis.

